# Supplementary material for: Geographic genetic variation in the Coral Hawkfish, Cirrhitichthys oxycephalus (Cirrhitidae), in relation to biogeographic barriers across the Tropical Indo-Pacific
Source: PeerJ. 2024 Sep 25;12:e18058. doi: 10.7717/peerj.18058 (PMC11438443; doi:10.7717/peerj.18058)
Supplement: Supplemental Information 3 [file peerj-12-18058-s003.pdf]

Geographic genetic variation in the Coral Hawkfish, *Cirrhitichthys oxycephalus* (Cirrhitidae), in relation to biogeographic barriers across the Tropical Indo-Pacific

ELECTRONIC SUPPLEMENTARY MATERIAL

Figure S1. IUCN Geographic range of *C. oxycephalus***.** Image taken from Greenfield, D. & Williams, I. 2016. *Cirrhitichthys oxycephalus* (errata version published in 2017). The IUCN Red List of Threatened Species 2016: e.T67997668A115451295. https://dx.doi.org/10.2305/IUCN.UK.2016-1.RLTS.T67997668A68001661.en

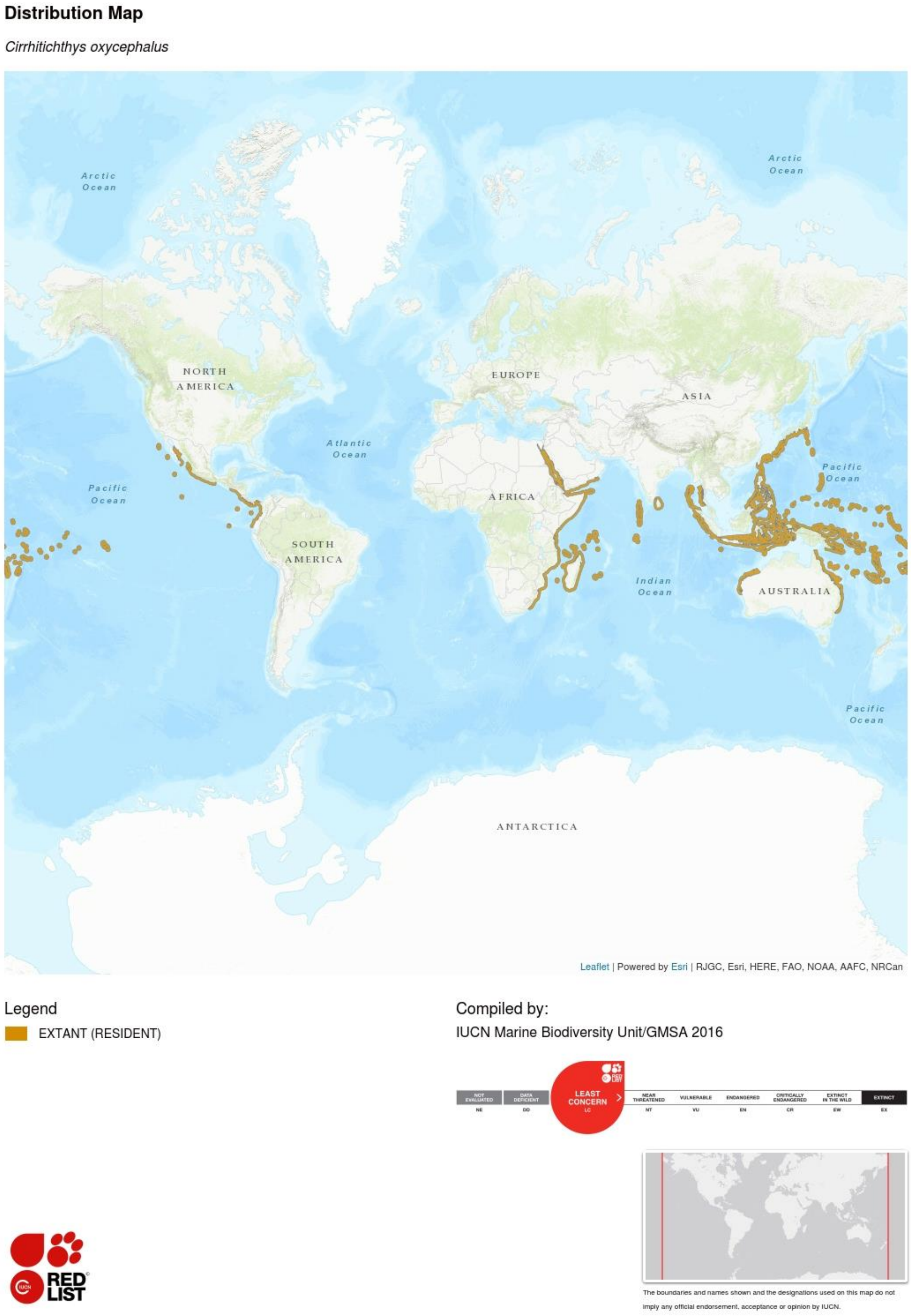

Figure S2. The phylogenetic tree based on the *RAG1* gene and ML. Posterior probability supports are shown in the nodes. Colors represent the different haplogroups (Hg) found in the haplotype network (Fig. 3). TEP: Tropical Eastern Pacific region except for CL-TEP: Clipperton-TEP.

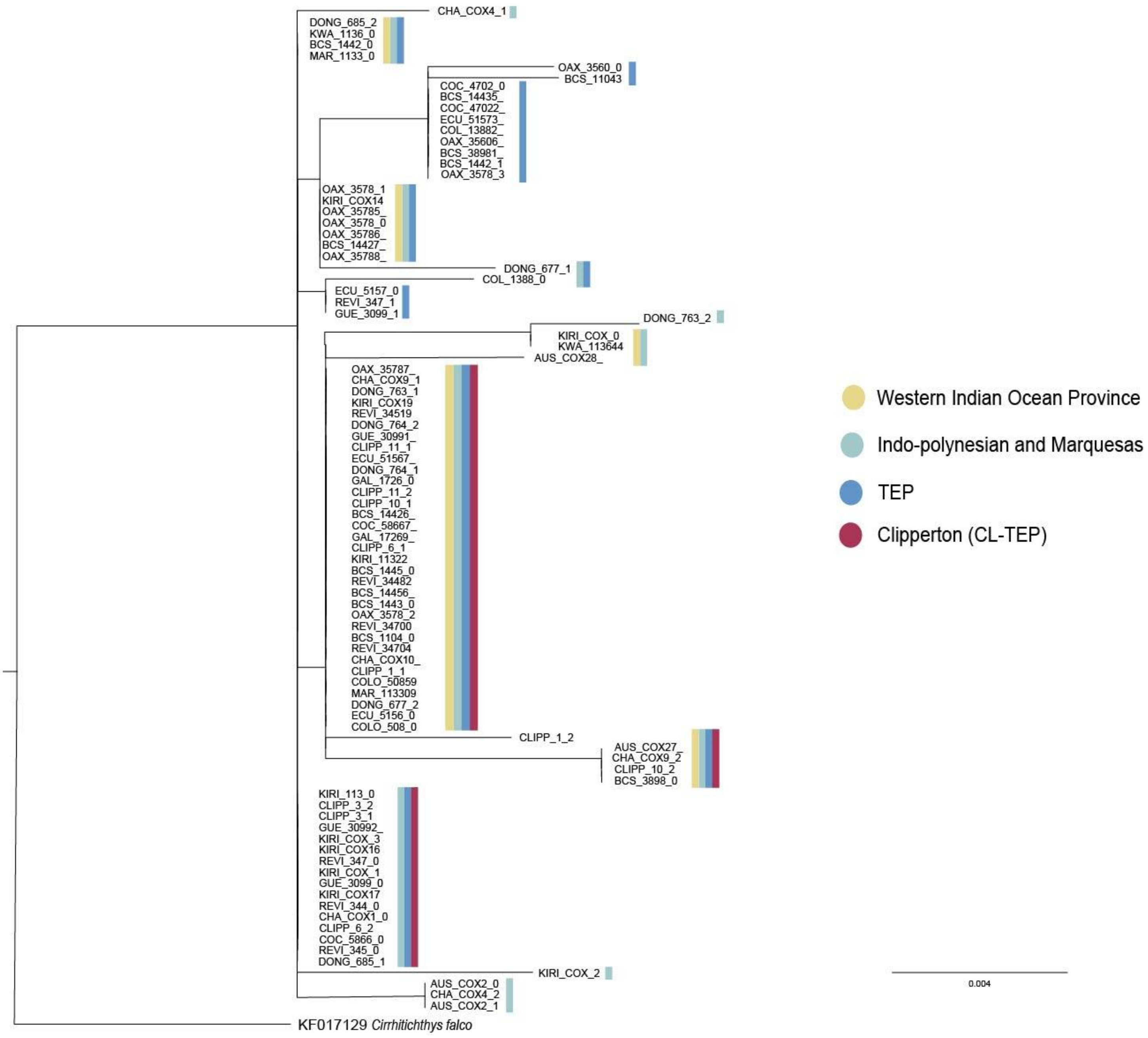

Table S1. Sample localities and associated information. CPUM: Fish collection of the Universidad Michoacana de San Nicolás de Hidalgo; UCF: University of Central Florida; KAUST: King Abdullah University of Science and Technology; Smithsonian: National Museum of Natural History of the Smithsonian Institute; UCSB: University of California Santa Barbara and HCMCUS: Ho Chi Minh City University of Science. N. *cytb*: number of specimens sequences for *cytb* gene; N. *RAG1*: number of specimens sequenced for *RAG1* gene.

Area/Country	Locality	Coordenates	Collection	N. *cytb*	ID Tissue	GenBank accession numbers	N. *RAG1*	ID Tissue
Saudi Arabia	Farasan Banks	18º 5´52.31" N 41º 18´38.35" E	UCF	1	ARA_COX11	PP328592	0	--
	Thuwal	22°14'15.73" N 39° 0'27.67" E	KAUST	1	ARA_COX6789	PP328593	0	--
	Al Lith	19°50.527' N 39°55.941 'E	KAUST	2	ARA_COX7741	PP328594	0	--
					ARA_COX7752	PP328595	0	--
South Africa	Durban, KwaZulu-Natal	29º 51´36" S 31º 3´35.9" E	Smithsonian	2	KWA_113041	PP328596	0	--
					KWA_113044	PP328597	0	--
	Sodwana, KwaZulu-Natal	27º 33´0" S 32º 41´23.9" E	Smithsonian	2	KWA_113644	PP328598	0	KWA_113644
					KWA_113645	PP328599	1	--
France	Reunion Island	21º 7´58.8" S 55º 32´16.80" E	Smithsonian	3	REU_113201	PP328600	0	--
					REU_113202	PP328601	0	--
					REU_113203	PP328602	0	--
Chagos Archipelago, United Kingdom	Egmont Atoll	6º 41´26.18" S 71º 50´12.67"E	UCF	2	CHA_COX4	PP328603	1	CHA_COX4
					CHA_COX5	PP328604	0	0
	Salomon Atoll	5º 13´57.16" S 72º 22´25.71"E	UCF	5	CHA_COX2	PP328605	0	0
					CHA_COX3	PP328606	0	0
					CHA_COX6	PP328607	0	0
					CHA_COX7	PP328608	0	0
					CHA_COX8	PP328609	0	0
	Great Chagos Bank	6º 41´26.18" S 71º 50´12.67"E	UCF	3	CHA_COX1	PP328610	0	0
					CHA_COX9	PP328611	2	CHA_COX9
					CHA_COX10	PP328612	0	CHA_COX10
Australia	Christmas Island	10º 26´22.96" S 105º 38´9.41" E	UCF	3	AUS_COX27	PP328613	2	AUS_COX27
					AUS_COX28	PP328614	0	AUS_COX28
					AUS_COX29	PP328615	0	0
	Great Barrier Reef	16º 51´0" S 145º 46´12" E	Smithsonian	1	AUS_113045	PP328616	0	0

Vietnam	No date	No date	HCMCUS	3	VIET_3	PP328617	0	0
					VIET_4	PP328618	0	0
					VIET_5	PP328619	0	0
Taiwan	Dongsha Atoll	20º 42´9.72" N 116º 48´28" E	UCF	19	DONG_468	PP328620	0	0
					DONG_671	PP328621	0	0
					DONG_672	PP328622	0	0
					DONG_673	PP328623	0	0
					DONG_674	PP328624	0	0
					DONG_675	PP328625	0	0
					DONG_676	PP328626	0	0
					DONG_677	PP328627	4	DONG_677
					DONG_678	PP328628	0	0
					DONG_679	PP328629	0	0
					DONG_680	PP328630	0	0
					DONG_684	PP328631	0	0
					DONG_685	PP328632	0	DONG_685
					DONG_763	PP328633	0	DONG_763
					DONG_764	PP328634	0	DONG_764
					DONG_778	PP328635	0	0
					DONG_782	PP328636	0	0
					DONG_783	PP328637	0	0
					DONG_784	PP328638	0	0
Kiribati	Kiritimati	1º 52´ 12" N 157º 18´36" W	Smithsonian	1	0	--	5	KIRI_113223
					KIRI_113225	PP328639	0	0
					0	--	0	0
		1º 57´ 0.238" N 157º 31´19.07" W	UCF	9	KIRI_COX13	PP328640	0	0
					KIRI_COX14	PP328641	0	KIRI_COX14
					KIRI_COX15	PP328642	0	0
					KIRI_COX16	PP328643	0	KIRI_COX16
					0	--	0	KIRI_COX17
					KIRI_COX18	PP328644	0	0
					KIRI_COX19	PP328645	0	KIRI_COX19
					KIRI_COX22	PP328646	0	0
					KIRI_COX24	PP328647	0	0

					KIRI_COX25	PP328648	0	0
French Polynesia	Ua-huka, Marquesas Island	8º 54´0" S 139º 33´0" W	Smithsonian	2	0	--	1	MAR_113309
					MAR_113310	PP328649	0	0
					MAR_113311	PP328650	0	0
Revillagigedo Archipelago, Mexico	North beach bay, Socorro Island	18º 51´36.66´´N 110º 59´4.82" W	CPUM	21	REVI_34482	PP328651	4	REVI_34482
					REVI_34483	PP328652	0	0
					REVI_34484	PP328653	0	0
					REVI_34518	PP328654	0	0
					REVI_34519	PP328655	0	REVI_34519
					REVI_34665	PP328656	0	0
					REVI_34694	PP328657	0	0
					REVI_34696	PP328658	0	0
					REVI_34697	PP328659	0	0
					REVI_34700	PP328660	0	REVI_34700
					REVI_34704	PP328661	0	REVI_34704
					REVI_34720	PP328662	0	0
					REVI_34721	PP328663	0	0
					REVI_34722	PP328664	0	0
					REVI_34792	PP328665	0	0
					REVI_34796	PP328666	0	0
					REVI_34798	PP328667	0	0
					REVI_34803	PP328668	0	0
					REVI_34804	PP328669	0	0
					REVI_41125	PP328670	0	0
					REVI_41126	PP328671	0	0
Mainland Mexico	Concepción Bay, BCS	26º 53´ 39.2" N 111º 49´ 25.6" W	CPUM	1	BCS_14456	PP328672	1	BCS_14456
	De los Sueños Bay BCS	23º 59´37.1" N 109º 49´32.5" W	CPUM	2	BCS_15232	PP328673	0	0
					BCS_38981	PP328674	1	BCS_38981
	Tortuga Island, BCS	27º 25´50.3" N 111º 51´58.3" W	CPUM	2	BCS_11043	PP328675	1	BCS_11043
					BCS_14600	PP328676	0	0
	Espíritu Santo Island, BCS	24º 29´9.78" N 110º 24´2.03" W	CPUM	2	BCS_15585	PP328677	0	0
					BCS_15586	PP328678	0	0
	Punta Pulpito, BCS	26º 30´52.3" N 111º 26´ 35.2" W	CPUM	4	BCS_14423	PP328679	0	0
					BCS_14426	PP328680	3	BCS_14426

					BCS_14427	PP328681	0	BCS_14427
					BCS_14435	PP328682	0	BCS_14435
	Punta Carrizales, Colima	19º 5´47.8" N<br>104º 26´20.56" W	CPUM	3	COL_13881	PP328683	1	0
					COL_13882	PP328684	0	COL_13882
					COL_13934	PP328685	0	0
	Morros del Potosí, Guerrero	17º 32´2.8" N<br>101º 29´50.4" W	CPUM	1	GUE_19074	PP328686	0	0
	Zacatoso,Guerrero	17º 39´ 14.33" N<br>101º 37´ 20.53" W	CPUM	1	GUE_19131	PP328687	0	0
	La Pedregosa, Guerrero	17º 37´ 20.4" N<br>101º 31´ 19.89" W	CPUM	2	GUE_30991	PP328688	2	GUE_30991
					GUE_30992	PP328689	0	GUE_30992
	Punta Chahue, Oaxaca	15º 45´9.97" N<br>96º 7´27.39" W	CPUM	7	OAX_35605	PP328690	1	0
					OAX_35606	PP328691	0	OAX_35606
					OAX_35607	PP328692	0	0
					OAX_35785	PP328693	4	OAX_35785
					OAX_35786	PP328694	0	OAX_35786
					OAX_35787	PP328695	0	OAX_35787
					OAX_35788	PP328696	0	OAX_35788
El Salvador	Los Cobanos, Sonsonate	13º 31´51.2" N<br>89º 50´48.1" W	CPUM	5	SALV_24377	PP328697	0	0
					SALV_24378	PP328698	0	0
					SALV_24409	PP328699	0	0
					SALV_24529	PP328700	0	0
					SALV_24538	PP328701	0	0
Mainland Costa Rica	Cabo Blanco, Puntarenas	9º 32´26.21" N<br>85º 6´40.35" W	CPUM	1	CR_39990	PP328702	0	0
Coco Island, Costa Rica	Punta María, Coco island	5º 32´7.3" N<br>87º 5´12.5" W	CPUM	3	COC_47020	PP328703	2	0
					COC_47021	PP328704	0	0
					COC_47022	PP328705	0	COC_47022
	Wafer bay, Coco island	5º 30´36" N<br>87º 3´36" W	Smithsonian	2	COC_110215	PP328706	0	0
			CPUM		COC_58667	PP328707	0	COC_58667
	Manuelita, Coco island	5° 33´37.67" N<br>87º 2´48.73" W	CPUM	4	COC_58707	PP328708	0	0
					COC_58708	PP328709	0	0
					COC_58709	PP328710	0	0
					COC_58737	PP328711	0	0
Panamá	Los Frailes, Darien	10º 17´24" N<br>80º 2´59.99" W	Smithsonian	3	PAN_113794	PP328712	0	0
					PAN_113795	PP328713	0	0

					PAN_113925	PP328714	0	0
	Los Frailes, Santos	7º 20´51.6" N<br>80º 8´11.5" W	CPUM	3	PAN_22317	PP328715	0	0
					PAN_22318	PP328716	0	0
					PAN_22319	PP328717	0	0
Colombia	Piedra Wacha, Choco	5º 36´19.7" N<br>77º 29´52.2" W	CPUM	2	COLO_50859	PP328718	1	COLO_50859
					COLO_50860	PP328719	0	0
Mainland Ecuador	Ureles, Manabí	1º 15´32.62" N<br>81º 4´20.62" W	CPUM	7	ECU_51191	PP328720	2	0
					ECU_51192	PP328721	0	0
					ECU_51563	PP328722	0	0
					ECU_51565	PP328723	0	0
					ECU_51567	PP328724	0	ECU_51567
					ECU_51569	PP328725	0	0
					ECU_51573	PP328726	0	ECU_51573
France	Clipperton Atoll	10º 18´14.4" N<br>109º 13´4.8" W	UCSB	4	CLIPP_1	PP328727	5	CLIPP_1
					CLIPP_4	PP328728	0	0
					CLIPP_5	PP328729	0	CLIPP_3
					CLIPP_9	PP328730	0	0
					0		0	CLIPP_6
					0		0	CLIPP_10
					0		0	CLIPP_11
			Smithsonian	15	CLIP_110373	PP328731	0	0
					CLIP_110459	PP328732	0	0
					CLIP_110460	PP328733	0	0
					CLIP_110461	PP328734	0	0
					CLIP_110462	PP328735	0	0
					CLIP_110463	PP328736	0	0
					CLIP_110464	PP328737	0	0
					CLIP_110465	PP328738	0	0
					CLIP_110466	PP328739	0	0
					CLIP_110467	PP328740	0	0
					CLIP_110468	PP328741	0	0
					CLIP_110469	PP328742	0	0
					CLIP_110470	PP328743	0	0
					CLIP_110471	PP328744	0	0

					CLIP_113383	PP328745	0	0
Galapagos Archipelago, Ecuador	Rocas Daphne,	0º 25´27.71" N<br>90º 21´30.4" W	CPUM	3	GAL_18395	PP328746	1	0
					GAL_18408	PP328747	0	0
					GAL_18438	PP328748	0	0
	Barco Hundido Karagua	0º 53´40.2" N<br>89º 37´2.19" W	CPUM	2	GAL_17269	PP328749	0	GAL_17269
					GAL_25476	PP328750	0	0
	Islote Mosquera,	0º 24´17.1" N<br>90º 16´32.4" W	CPUM	3	GAL_17483	PP328751	0	0
					GAL_17484	PP328752	0	0
					GAL_17485	PP328753	0	0
	León Dormido	0º 46´42.76" N<br>89º 31´18.3" W	CPUM	3	GAL_17575	PP328754	0	0
					GAL_17576	PP328755	0	0
					GAL_25460	PP328756	0	0
	Roca Ballena	0º 56´48.01" N<br>89º 34´52.1" W	CPUM	1	GAL_17404	PP328757	0	0
			**Total**	166			45	

Table S2. Above diagonal pairwise population comparison (ΦST) and below diagonal mean genetic distances (D_P) According with location/regions for *cytb* and *RAG1* gene.

Gene		Arabia	South Africa	Christmas	Vietnam	Dongsha	Australia	Kiribati	Chagos	Marquesas	Revilla	Cortez	Mexican	Panamic	Cocos	Clipperton	Galapagos
	Arabia		-0.029	-0.157	-0.018	0.118	-0.569	0.073	0.018	-0.054	0.583*	0.671*	0.497*	0.715*	0.63*	0.747*	0.719*
	S.Africa	1.783		-0.036	0.022	0.077	-0.268	0.126*	0.032	0.065	0.543*	0.609*	0.468*	0.657*	0.566*	0.696*	0.643*
	Christmas	1.562	1.672		-0.043	0.163	-0.684	0.081	0.069	-0.02	0.604*	0.715*	0.512*	0.746*	0.678*	0.773*	0.769*
	Vietnam	1.404	1.440	1.207		0.032	-0.6	-0.011	0.003	0.117	0.621*	0.76*	0.532*	0.769*	0.724*	0.801*	**0.818***
	Dongsha	1.515	1.448	1.448	0.975		-0.125	0.059	0.027	0.173	0.599*	0.658*	0.558*	0.681*	0.638*	0.716*	0.68*
	Australia	1.22	1.395	0.997	0.525	0.887		-0.411	-0.23	-0.2	0.597*	0.782	0.465	0.785*	0.731	0.82*	0.859
	Kiribati	1.409	1.448	1.276	0.861	1.007	0.630		0.005	-0.007	0.623*	0.71*	0.568*	0.732*	0.688*	0.762*	0.746*
cytb	Chagos	1.457	1.458	1.396	0.997	1.068	0.882	0.992		-0.005	0.589*	0.67*	0.533*	0.7*	0.645*	0.728*	0.703*
	Marquesas	1.496	1.642	1.365	0.997	1.194	0.787	0.898	1.039		0.634*	0.781*	0.538*	0.79*	0.747*	0.805*	0.841*
	Revilla	2.972	2.783	2.910	2.612	2.524	2.542	2.587	2.553	2.775		0.03	-0.011	0.039	0.026	0.426*	0.066*
	Cortez	2.795	2.638	2.715	2.424	2.355	2.348	2.388	2.385	2.591	0.805		0.002	0.001	0.077	0.646*	0.072*
	Mexican	**3.228**	3.021	3.161	2.868	2.773	2.801	2.846	2.818	3.048	1.241	1.016		0.017	-0.019	0.425*	0.028
	Panamic	2.814	2.624	2.745	2.435	2.342	2.392	2.418	2.396	2.640	0.800	0.512	1.009		0.082*	0.64*	0.01
	Cocos	2.865	2.655	2.817	2.496	2.397	2.380	2.471	2.457	2.712	0.869	0.620	1.064	0.621		0.626*	0.212*
	Clipperton	3.081	2.909	2.956	2.688	2.612	2.677	2.657	2.598	2.702	1.324	1.392	1.650	1.385	1.441		0.675*
	Galapagos	2.690	2.510	2.612	2.340	2.259	2.323	2.310	2.281	2.507	0.743	0.450	0.948	0.428	0.602	1.287	
	Arabian		--	--	--	--	--	--	--	--	--	--	--	--	--	--	--
	SouthAfrica	--		0.111	--	-0.227	--	-0.147	-0.304	-0.500	-0.039	0.043	0.179	-0.058	0.055	0.016	0
	Christmas	--	1.515		--	0.205*	--	0.254**	0.021	0.040	0.259	0.214	0.317	0.172	0.222	0.295*	0.245
	Vietnam	--	--	--		--	--	--	--	--	--	--	--	--	--	--	--
	Dongsha	--	1.042	1.515	--		--	0.001	-0.074	-0.350	-0.047	0.083	0.214*	-0.038	0.144	-0.037	-0.192
	Australia	--	--	--	--	--		--	--	--	--	--	--	--	--	--	--
RAG 1	Kiribati	--	0.985	1.439	--	1.061	--		-0.041	-0.308	-0.023	0.119	0.156*	0.073	0.122	0.096	0.141
	Chagos	--	1.010	1.263	--	1.105	--	1.035		-0.384	-0.105	0.046	0.174*	-0.080	0.070	-0.037	-0.09
	Marquesas	--	0.758	1.136	--	0.758	--	0.682	0.758		-0.416	-0.092	0.073	-0.263	-0.029	-0.301	0
	Revilla	--	0.852	1.184	--	0.852	--	0.777	0.821	0.473		0.078	0.209*	-0.055	0.177	-0.055	0.053
	Cortez	--	1.452	1.673	--	1.373	--	1.313	1.347	1.073	1.089		0.007	-0.066	-0.136	0.130	0.074
	Mexican	--	1.563	**1.799**	--	1.515	--	1.297	1.468	1.184	1.207	1.286		0.123	-0.158	**0.298*** **	0.319
	Panamic	--	1.136	1.389	--	1.073	--	1.086	1.052	0.758	0.789	1.136	1.310		-0.008	-0.047	-0.135
	Cocos	--	1.515	1.705	--	1.468	--	1.288	1.389	1.136	1.136	1.199	1.113	1.199		0.278	0.284

Clipperton	--	0.909	1.250	--	0.871	--	0.894	0.884	0.530	0.606	1.162	1.373	0.808	1.288		-0.189
Galapagos	--	0.758	1.136	--	0.663	--	0.833	0.758	0.379	0.473	1.073	1.420	0.631	1.326	0.379	

Note. One asterisk = $p < 0.05$, two asterisks = $p < 0.01$ and three asterisks = $p < 0.001$. Revilla = Revillagigedo. Highest values highlighted in bold text.

Table S3. Above diagonal pairwise comparison (ΦST) and below diagonal mean genetic distances (D_p) in percentage for cy*tb* and *RAG1* gene. According with the regionalization presented by Briggs & Bowen (2012).

Gene	Region	RSP	WIOP	IPHMEIP	TEP
cytb	Red Sea Province (RSP)		-0.029	0.074	0.617***
	Western Indian Ocean Province (WIOP)	1.783		0.092*	0.586***
	Indo-Polynesian and Marquesas Provinces (IP-M)	1.47	1.479		0.606***
	Tropical Eastern Pacific	2.935	2.749	2.537	
RAG1	Red Sea Province		--	--	--
	Western Indian Ocean Province	--		-0.158	0.024
	Indo-Polynesian and Marquesas Provinces	--	1.061		0.061**
	Tropical Eastern Pacific	--	1.254	1.192	

Note. One asterisk = $p < 0.05$, two asterisks = $p < 0.01$ and three asterisks = $p < 0.001$.

Table S4. Diversity indices of mitochondrial *cytb* and nuclear *RAG1* genes. According with location/regions.

cytb	*N*	*hn*	*SS*	*h*	*π*
Arabian	4	4	24	1 +/- 0.177	0.019 +/-0.004
South Africa	7	7	36	1 +/- 0.076	0.018 +/- 0.002
Christmas	3	3	17	1 +/- 0.272	0.017 +/- 0.005
Vietnam	3	2	9	0.667 +/- 0.314	0.009 +/- 0.004
Dongsha	19	16	32	0.977 +/- 0.027	0.010 +/- 0.001
Australia	1	0	0	0	0
Kiribati	10	9	22	0.978 +/- 0.054	0.009 +/- 0.001
Chagos	10	10	31	1 +/- 0.045	0.011 +/- 0.001
Marquesas	2	2	6	1 +/- 0.500	0.009 +/- 0.007
Revillagigedo	21	17	33	0.976 +/- 0.023	0.010 +/- 0.001
Cortez	11	9	12	0.964 +/- 0.051	0.005 +/- 0.001
Mexican	14	13	53	0.989 +/- 0.031	0.015 +/- 0.005
Panamic	21	16	24	0.929 +/- 0.051	0.005 +/- 0.001
Cocos	9	6	10	0.880 +/- 0.091	0.006 +/- 0.001
Clipperton	19	7	25	0.754 +/- 0.071	0.005 +/- 0.002
Galapagos	12	9	13	0.909 +/- 0.079	0.004 +/- 0.001
RAG1					
Arabian	0	0	0	0	0
South Africa	2	2	2	1 +/- 0.500	0.015 +/- 0.008
Christmas	4	3	3	0.049 +/- 0.222	0.013 +/- 0.005
Vietnam	0	0	0	0	0
Dongsha	8	4	5	0.75 +/- 0.139	0.012 +/- 0.004
Australia	0	0	0	0	0
Kiribati	10	5	5	0.756 +/- 0.130	0.0096 +/- 0.003
Chagos	6	5	5	0.933 +/- 0.122	0.012 +/- 0.003
Marquesas	2	2	1	1 +/- 0.500	0.008 +/- 0.004
Revillagigedo	8	3	2	0.679 +/- 0.122	0.006 +/- 0.002
Cortez	12	6	4	0.803 +/- 0.096	0.013 +/- 0.002
Mexican	16	7	5	0.858 +/- 0.057	0.012 +/- 0.002
Panamic	6	3	3	0.0463 +/- 0.215	0.011 +/- 0.004
Cocos	4	3	3	0.833 +/- 0.222	0.014 +/- 0.004
Clipperton	10	4	3	0.711 +/- 0.117	0.007 +/- 0.002
Galapagos	2	1	0	0	0

Note. *N*, number of individuals per Haplogroup; *hn*, number of haplotypes; *SS*, segregating sites; *h*, haplotype diversity; *π*, Nucleotide diversity.
